# Mebendazole, an anti-helminth drug, suppresses inflammation, oxidative stress and injury in a mouse model of ulcerative colitis

**DOI:** 10.1038/s41598-022-14420-6

**Published:** 2022-06-17

**Authors:** Moein Eskandari, Fereshteh Asgharzadeh, Mohammad Mostafa Askarnia-faal, Hamideh Naimi, Amir Avan, Mitra Ahadi, Hassan Vossoughinia, Masoumeh Gharib, Atena Soleimani, Niloufar Naghibzadeh, Gordon Ferns, Mikhail Ryzhikov, Majid Khazaei, Seyed Mahdi Hassanian

**Affiliations:** 1 Department of Clinical Biochemistry, Faculty of Medicine, Mashhad University of Medical Sciences, Mashhad, Iran; 2 Department of Medical Physiology, Faculty of Medicine, Mashhad University of Medical Sciences, Mashhad, Iran; 3 Metabolic Syndrome Research Center, Mashhad University of Medical Sciences, Mashhad, Iran; 4 Department of Human Genetics, Faculty of Medicine, Mashhad University of Medical Sciences, Mashhad, Iran; 5 Department of Gastroenterology and Hepatology, Faculty of Medicine, Mashhad University of Medical Sciences, Mashhad, Iran; 6 Department of Pathology, Faculty of Medicine, Mashhad University of Medical Sciences, Mashhad, Iran; 7 Brighton & Sussex Medical School, Division of Medical Education, Falmer, Brighton, BN1 9PH Sussex UK; 8 School of Medicine, Saint Louis University, Saint Louis, MO USA

**Keywords:** Gastrointestinal diseases, Inflammatory bowel disease, Ulcerative colitis, Biochemistry, Gastroenterology

## Abstract

Mebendazole (MBZ) is an efficacious anthelmintic with known anti-inflammatory and fibrinolytic properties. In this study, we aimed to explore the protective effects of this FDA-approved drug against DSS-induced colitis in a murine model either alone or in combination with Sulfasalazine (SSZ), a standard therapy for ulcerative colitis. We found that MBZ significantly improved colitis disease activity index as assessed by changes in body weight, degree of stool consistency, rectal bleeding, and prolapse. We also found that MBZ ameliorated the colon histopathological score by attenuating crypt loss, mucosal damage, and inflammation score in colitis tissues. Similarly, DSS-induced colon shortening, colon weight loss, and increase in spleen weight were all abrogated in the presence of MBZ. Moreover, MBZ decreased inflammation, possibly by reducing oxidative stress markers, suppressing inflammatory cell infiltration, and down-regulation of inflammatory genes in colon tissues. Furthermore, MBZ potently reduced fibrosis by decreasing collagen deposition and down-regulating pro-fibrotic genes including Col 1a1 and Col 1a2 in colitis tissue homogenates. In conclusion, our study showed that this broad-spectrum anthelminthic could be repurposed as a novel therapy for ulcerative colitis without any observed side effects, however, regarding the concerns about the potential toxicity of MBZ in UC patients, future experiments on MBZ therapy in other models of UC is needed to completely address the toxicity concerns.

## Introduction

Ulcerative colitis (UC) is a sub-category of inflammatory bowel diseases (IBD) that causes mucosal inflammation in the rectum and lower colon^1^. Although the exact pathogenesis of ulcerative colitis remains unclear, potential risk factors include altered immune responses, overactive immune response toward commensal microflora, genetic susceptibility, and environmental factors that have been considered as potential risk factors for UC^2^. Clinical manifestations of UC consist of abdominal pain, bloody diarrhea, weight loss, and anemia caused by mucosal damage. Endoscopic and histologic evaluation along with clinical assessment is used to diagnose UC^3^. Chronic ulcerative colitis can precede colorectal cancer, with chronic UC patients facing a higher risk of developing colitis-associated colorectal cancer (CAC)^2^.

Inflammation and fibrosis play pivotal roles in the pathogenesis of colitis. Recent findings support the importance of mucosal healing and regulation of inflammatory response in colitis^2,4^. In ulcerative colitis, inflammation and lesions are restricted to the mucosal layer causing superficial damage to the endothelial tissues of the colon^1^. Moreover, in ulcerative colitis, a dysregulated immune response modulates the gut environment by enhancing the excessive secretion of multiple pro-inflammatory factors^5^ including platelet-derived growth factor, interleukin-13 (IL-13), and IL-17^6^. Tissue fibrosis is a common consequence of chronic inflammation as a major response to inflammation of damaged tissue characterized by excess accumulation of a collagen-rich extracellular matrix (ECM)^7^. Fibrotic mediators such as transforming growth factor-beta (TGF-β) activate the mesenchymal cells to migrate to the inflamed tissues and transform into activated myofibroblasts. Myofibroblasts are able to produce large amounts of ECM^8^. ECM accumulation and deposition of excess collagen are common features of colitis.

Therapies targeting inflammation and fibrosis are now among the most widely used drugs for UC treatment. Sulfasalazine (SSZ) is one of the current standard therapeutic agents widely administrated orally for UC treatment^9^. Although sulfasalazine attenuates inflammation by inhibiting prostaglandin synthesis in the gut^10^, it is reported to cause various side effects in patients^11^. Thus, it is crucial to find a new form of therapy with fewer adverse effects and improved clinical remission and mucosal healing^12,13^.

Methyl-5-benzoyl-2-benzimidazole carbamate (Mebendazole, MBZ) is an FDA-approved Benzimidazole that shows efficacy against a broad spectrum of intestinal helminthiasis^14^ and can be safely administrated to children^15^ and adults^16^. The safety and limited cytotoxicity of mebendazole has been demonstrated in several clinical trials of approximately 6300 patients^17^. MBZ elicits anti-inflammatory and anti-fibrotic effects in different cell lines and animal models via down-regulation of the mitogen-activated protein kinase (MAPK)^18,19^, nuclear factor- kappa B (NF-κB)^20^, cyclooxygenase 2 (COX2)^21^ and TGF-β signaling pathways^19^. It also acts as an anti-fibrotic by reducing alpha-smooth muscle actin (α-SMA) levels^22^ and collagen release from cells^23^. We aimed to evaluate the therapeutic efficacy of mebendazole in a murine DSS-induced colitis model either alone or in combination with sulfasalazine, a standard therapy for UC.

##  Material and methods

### Material

Dextran sulfate sodium (DSS-40 kDa) was purchased from Sigma-Aldrich (St. Louis, MO). Mebendazole (100 mg) and sulfasalazine (500 mg) tablets were also obtained from Sigma-Aldrich (St. Louis, MO). IL-6 and TNF-α ELISA kits were purchased from Elabscience Biotechnology Co., Ltd., Wuhan, China. Ketamine 10% was purchased from Medistar (Ascheberg, Germany) and Xylazine 2% was obtained from Riemser (Greifswald, Germany).

### Animals

Inbred 8-week-old C57BL/6 mice were purchased from the Pasteur Institute of Iran (Tehran, Iran). The animals were maintained according to a standard protocol of Institutional Animal Care Guidelines with standard housing conditions (Temperature 22–25 °C; Humidity 55–60%), a 12 h light/dark cycle, and ad libitum access to food and water ad libitum. Animal experiments were conducted according to the ARRIVE guidelines and guidelines for Care and Use of Laboratory Animals from the Mashhad University of Medical Sciences ethics committee. The study was approved by the ethics committee of Mashhad University of Medical Sciences (Approval ID: IR.MUMS.MEDICAL.REC.1400.112).

### Colitis model and experimental design

**Figure 1:**
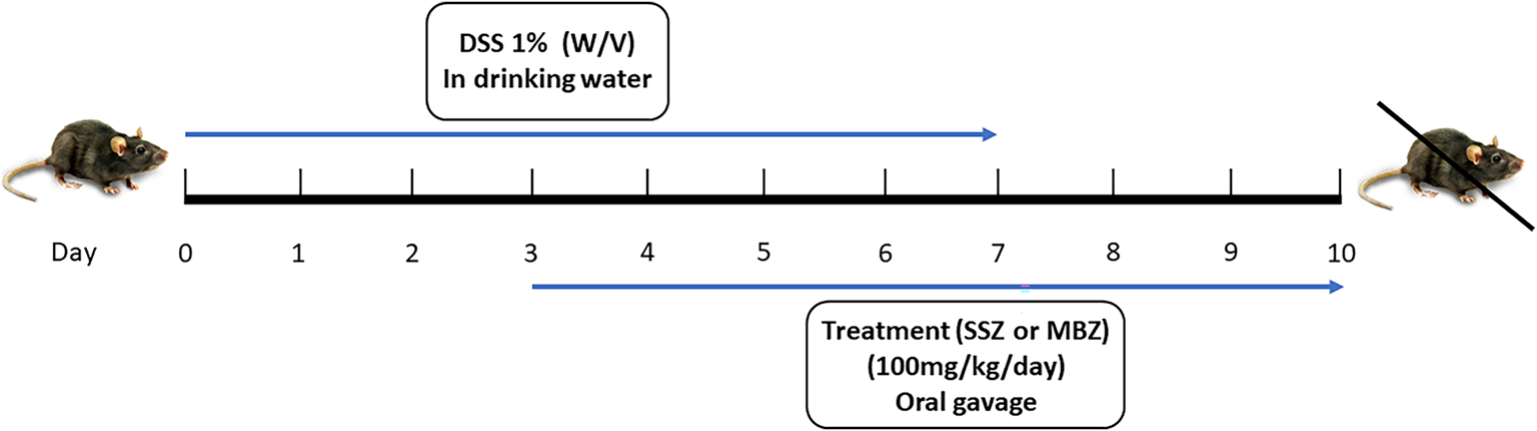
Schematic presentation of the study design.

The experimental animals were randomly assigned to five groups. Group 1 or the control group (normal) was given drinking water alone for 10 days (n = 6). The other four groups were treated with Dextran Sodium Sulfate (DSS) solution (1%) to induce UC-like symptoms in experimental animals**.** Group 2, the colitis group was given DSS solution for the first 7 days and normal drinking water on days 7–10 (n = 6). Group 3, the Sulfasalazine group was given DSS solution for the first 7 days and sulfasalazine (100 mg/kg/day, oral gavage) from days 3–10 (n = 6). Group 4 or the Mebendazole group was given DSS solution for the first 7 days and mebendazole (100 mg/kg/day, oral gavage)^19,24–26^ from day 3–10 (n = 6). Group 5, the combination group was given DSS solution for the first 7 days and sulfasalazine (100 mg/kg/day, oral gavage) and mebendazole (100 mg/kg/day, oral gavage) days 3–10 (n = 6). A schematic figure of the treatment of murine colitis model is presented in Fig. 1.

During the experiment, the disease activity index (DAI), which consists of 4 parameters as described in Table 1 was evaluated daily. At the end of the experiment, animals were anesthetized by intraperitoneal injection of ketamine and xylazine mixture to the right lower quadrant of the abdomen using an insulin syringe and then sacrificed with the cervical dislocation method. After sacrifice, colon, and spleen were collected, weighed and length was recorded. Tissues were fixed in 10% formalin or kept in liquid nitrogen for subsequent investigations.

**Table 1:** The Ulcerative Colitis Disease Activity Index (UCDAI) scoring system.

Score	Rectal bleeding	Stool consistency	Rectal prolapse	Lose weight (%)
0	None	Normal	None	< 5
1	Red	Soft	Sign of prolapse	5–10
2	Dark red	Very soft	Clear prolapse	10–15
3	Gross bleeding	Diarrhea	Extensive prolapse	> 15

### Histopathological evaluation

For histological analysis, colon, heart, liver, kidney, and lung specimens were harvested and placed immediately in 10% buffered formalin. Tissue samples were processed and sectioned following paraffin embedding. Sections were stained with hematoxylin and eosin (H&E) or Masson's trichrome to visualize inflammatory or fibrotic lesions, respectively. Using light microscopy, tissues were scored according to the criteria described in Table 2. To further evaluate the side effects of mebendazole, heart, kidney, liver, and lungs of mice treated with mebendazole were also transferred in 10% buffered formalin.

**Table 2:** Histopathological criteria used for colorectal tissue damage scoring.

Score	0	1	2	3	4
Inflammation	None	Mild	Moderate	Severe	
Mucosal damage	None	Mucus layer	Submucosa	Muscular and serosa	
Crypt loss	None	1/3	2/3	100% + intact epithelium	100% with epithelium lose
Pathological change range	None	1–25%	26–50%	51–75%	76–100%

### Oxidative stress measurement

To evaluate the content of oxidative stress markers, the tissue concentrations of total thiol, malonyl dialdehyde (MDA), and the enzyme activity of superoxide dismutase (SOD) and catalase (CAT) were measured in colon tissues as described previously^27,28^.

### Real-time PCR

Samples were homogenized and total RNA was extracted using Total RNA Extraction mini kit (Favorgen, Taiwan). Using Easy cDNA synthesis kit (Pars Tous, Iran), complementary DNA (cDNA) was synthesized following the manufacturer’s instructions. Quantitative real‐time polymerase chain reaction (RT-PCR) was performed via the resulting cDNA as a template and amplification was carried out by the Ampliqon SYBR Green PCR Master Mix as previously described^29^. The mRNA expression of pro-inflammatory and pro-fibrotic genes was assessed using specific primers purchased from Macrogene Co. (Seoul, Korea) (Table 3). Glyceraldehyde ‐3 ‐phosphate dehydrogenase (GAPDH) was used as an internal control gene.

**Table 3:** qPCR primers sequence.

Gene	Source	Primer	Sequence
GAPDH	Mouse	Forward	CAACGACCCCTTCATTGACC
		Reverse	CTTCCCATTCTCGCCTTGA
Col1a1	Mouse	Forward	AATGGTGAGACGTGGAAACC
		Reverse	GACAGTCCAGTTCTTCATTGCA
Col1a2	Mouse	Forward	GTTCTCAGGGTAGCCAAGGT
		Reverse	CCTTCAAAACCAAAGTCATAGCC
IL-1β	Mouse	Forward	GACTTCACCATGGAATCCGT
		Reverse	TGCTCATTCACGAAAAGGGA

### ELISA assay

Tissue concentrations of IL-6 and TNF-α were evaluated in colon tissues of mice. Samples were homogenized in PBS according to manufacturer instructions. After homogenization, protein concentrations were measured with the BCA method. Samples were then diluted with PBS to reach a concentration of 2500 µg/ml, and tissue levels of IL-6 and TNF-α were measured using ELISA kit as described (Elabscience Biotechnology Co., Ltd., Wuhan, China).

### Liquid Chromatography–Mass Spectrometry (LC–MS)

To investigate the toxicity of mebendazole, plasma concentrations of mebendazole were measured using an LC/MS system. Standard samples of mebendazole were prepared at final concentrations of 5, 500, and 50 × 10^3^ ng/ml and administered intravenously. Blood was obtained from healthy and colitis mice which were treated with 100 mg/kg/day of mebendazole and plasma samples were prepared as previously described^30,31^. The Shimadzu UFLC LC-AD20 liquid chromatography system (Shimadzu, Japan) was equipped with a DGU-20A3R Degasser, a Binary pump (LC-20AD), an autosampler (SIL-20AC HT), and a column oven (CTO-20 AC). Analytes were separated with a SUPELCO analytical Discovery HS C18 column (150 mm × 4.6 mm, 3 µm, PA, USA). The temperature of the column oven was set at 40 °C. Isocratic elution program was applied with mobile phase consisting of 50% water/0.1% formic acid (phase A), and 50% acetonitrile/0.1% formic acid (phase B). The flow rate and injection volume were 0.2 mL/min and 3 µL, respectively. Analysis was performed using a 3200 QTRAP mass spectrometer instrument (AB Sciex, MA, USA) operated in positive (ESI+) electrospray ionization mode. Analysis was performed with nitrogen using the following setting: curtain gas supply was set at a pressure of 10 psi, ion source gas 1 with a pressure of 40 psi, and ion source gas 2 with a pressure of 40 psi. The source temperature and ion spray voltage (IS) were set at 500 °C and 5000 V, respectively. The mass spectrometer was operated in multiple reaction monitoring (MRM) mode. The mass spectrometer was operated at unit mass resolution for both Q1 and Q3 in MRM mode using a dwell time of 150 ms for all analytes. Fragmentation was induced with collision energy (CE) of 50 eV. The m/z transition was 296 for the protonated molecule to 105 for the fragment ion. Results were calculated using AB Sciex Analyst software (version 1.6.3).

### Statistical analysis

Statistical analysis was performed using one‐way ANOVA and the Wilcoxon Mann–Whitney tests with LSD posthoc test. Data are presented as mean ± standard error of the mean (SEM) with differences of p < 0.05 considered statistically significant. Data were obtained from three independent experiments.

##  Results

### Mebendazole attenuated ulcerative colitis-associated clinical symptoms in the mouse model

**Figure 2:**
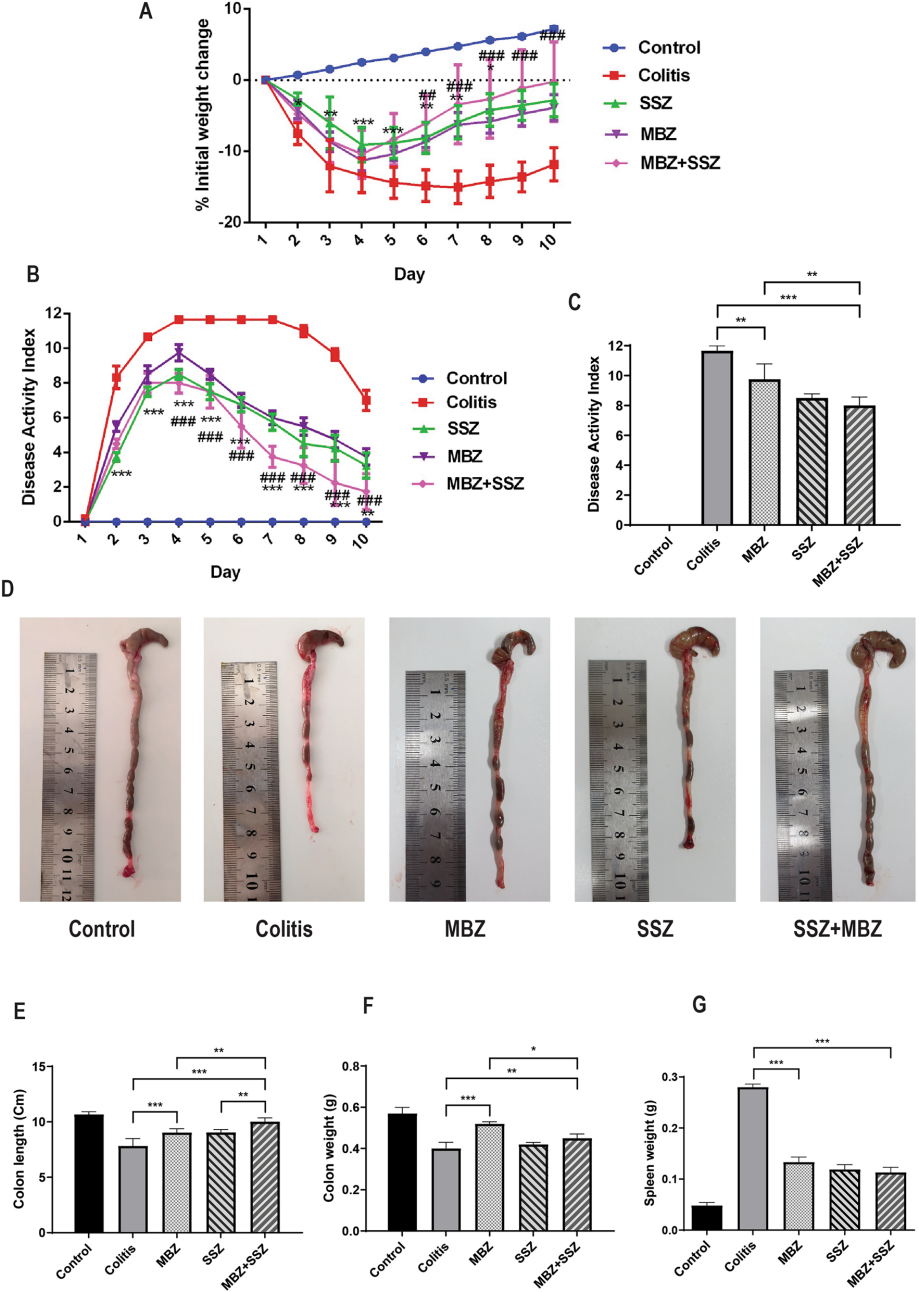
Efficacy of mebendazole on colitis clinical symptoms. (**A**) The effects of MBZ (100 mg/kg/day) alone or in combination with SSZ on body weight loss in DSS-treated mice. (**B**) The inhibitory effect of mebendazole on disease activity index at different time points is presented. (**C**) The highest DAI during the experiment period (10 days) is shown in each group. (**D**–**E**) MBZ efficacy against DSS-induced colon shortening was evaluated. (**F**) Colon weights and (**G**) spleen weights were measured in different groups. ** *P* < 0.01, *** *P* < 0.001. Data were presented as Mean ± SEM, DAI, colon weight and length, and spleen weight were analyzed by one-way ANOVA followed by post-hoc LSD test. Data are representative of three independent experiments with 6 mice in each group (n = 6).

Based on daily weight monitoring, animals showed a significant weight loss during DSS treatment. Administration of mebendazole (MBZ) or sulfasalazine improved body weight gain, which was more significant in the combination group (Fig. 2A). It is worth noting that the reduction in weight loss in the colitis group from day 7 to 10 is due to the replacement of DSS with drinking water in this period. Moreover, parameters of disease activity index (DAI) including stool consistency, rectal bleeding, and rectal prolapse were also compared between groups. The results showed that MBZ either alone or in combination with the standard treatment, sulfasalazine, significantly reduced DAI in the colitis mice (Fig. 2B–C). We also showed that MBZ decreased DSS-induced colon shortening (Fig. 2D–E), colon weight loss (Fig. 2F), and an increase in spleen weight (Fig. 2G). Colon weight loss and increase in spleen weight are markers of colon inflammation and splenic macrophage infiltration, respectively. These results showed the efficacy of MBZ alone or in combination with SSZ in relieving clinical symptoms of colitis in the mice model.

### Mebendazole attenuates inflammation in vivo

**Figure 3:**
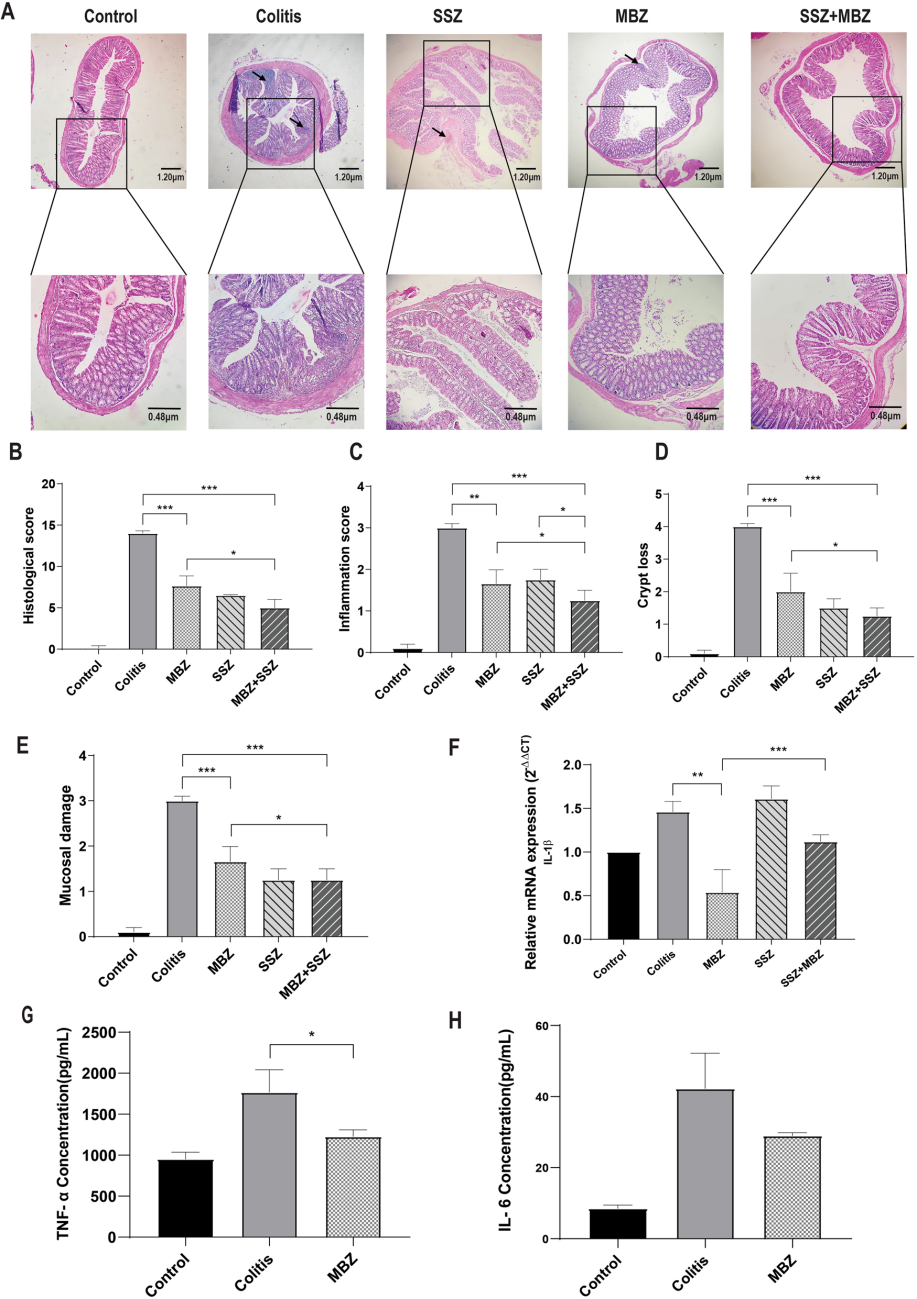
Mebendazole (100 mg/kg/day) reduced DSS-induced colon tissue inflammation. (**A**) Histologic sections of the colon are shown after undergoing hematoxylin and eosin (H&E) staining in the colitis tissues indicating the protective effect of MBZ or MBZ + SSZ on histological damages induced by DSS administration. Arrows indicate inflammatory cells infiltration. (**B**) Quantifying the effect of mebendazole on the histological score, (**C**) inflammation score, (**D**) crypt loss, and (**E**) mucosal damage in DSS-induced colitis mice. (**F**) Mebendazole significantly reduced the mRNA expression of IL-1β in colon tissue compared to the DSS group. (**G**) Mebendazole significantly reduced tissue concentrations of TNF-α protein, (**H**) The same as G, except that the effect of MBZ on IL-6 concentration was evaluated. * *P* < 0.05, ** *P* < 0.01, *** *P* < 0.001. Data were presented as Mean ± SEM, One-way ANOVA followed by post-hoc LSD test. Data are representative of three independent experiments with 6 mice in each group (n = 6).

Hematoxylin–eosin staining was performed to evaluate the effect of mebendazole on DSS-induced morphological changes and inflammation. Our results show the inhibitory effect of mebendazole alone or in combination with sulfasalazine on the infiltration of inflammatory cells into the affected tissue (Fig. 3A). Compared to the colitis group, the histopathological score (Fig. 3B), consisting of inflammation severity (Fig. 3C), crypt loss (Fig. 3D), and mucosal damage (Fig. 3E) was significantly decreased in mebendazole alone or combination-treated groups. In line with these results, mebendazole administration also caused a significant reduction in IL-1β mRNA expression in colitis tissues (Fig. 3F). Next, we compared levels of TNF-α and IL-6 in colitis mice in the presence and absence of MBZ using ELISA assay. Our results showed that mebendazole could decrease protein concentrations of these two pro-inflammatory markers in colitis tissue samples, however, only the decrease in TNF-α level was statistically significant (Fig. 3G–H). These results support the protective effects of MBZ on DSS-induced histological damage to colitis tissues.

### Mebendazole effects on oxidative stress markers

**Figure 4:**
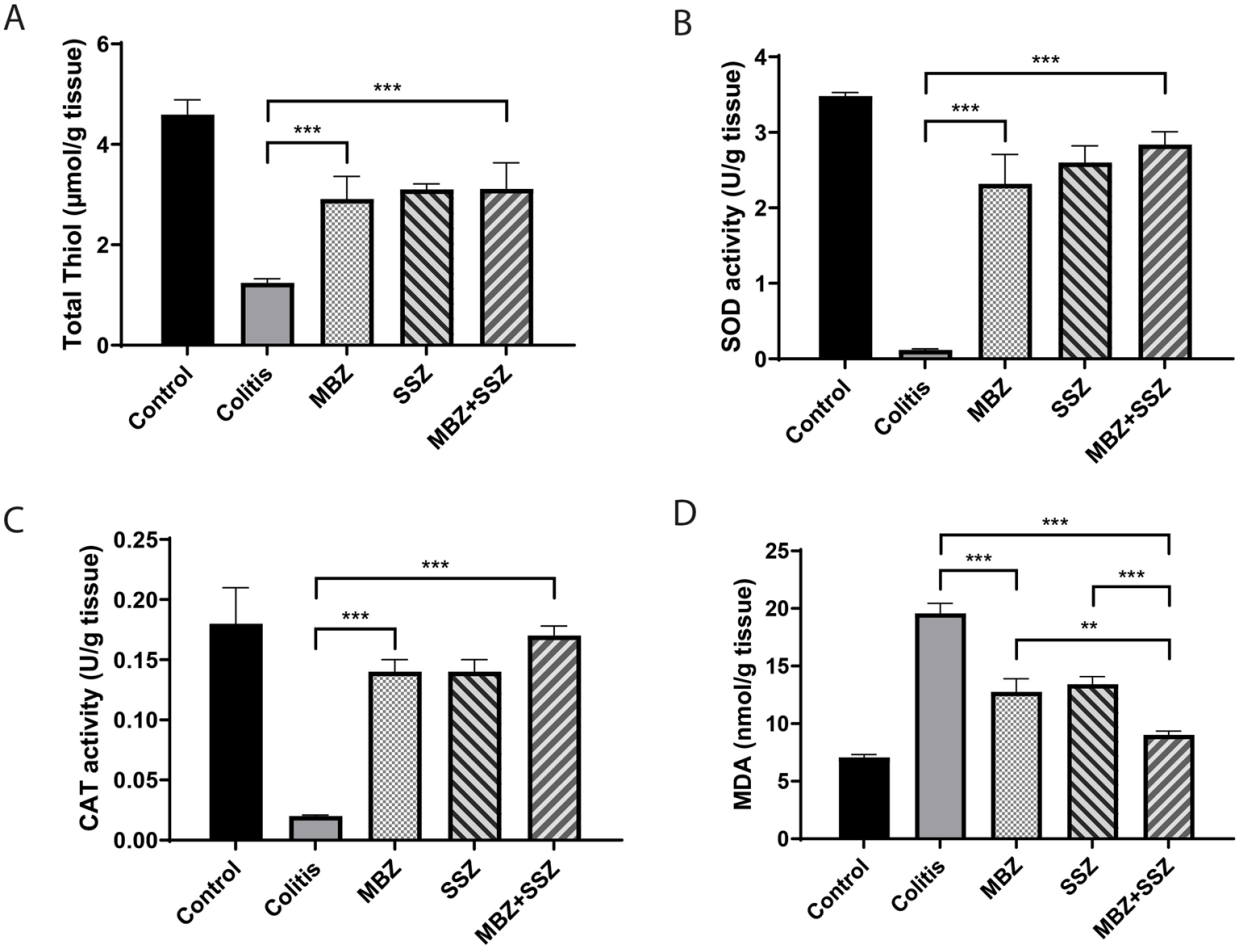
Anti-oxidant effect of mebendazole on colitis. (**A**) Total thiol content, (**B**) SOD activity, (**C**) CAT activity and (**D**) MDA Level, were compared between different groups. * *P* < 0.05, ** *P* < 0.01, *** *P* < 0.001. Data were presented as Mean ± SEM, One-way ANOVA followed by post-hoc LSD test. Data are representative of three independent experiments with 6 mice in each group (n = 6).

To further investigate the protective effects of mebendazole, we measured levels or activities of oxidative stress mediators in colon tissues. Compared to control colitis mice, treatment of colitis mice with MBZ suppressed the inhibitory effect of DSS on total thiol level (Fig. 4A) as well as SOD (Fig. 4B) and catalase (Fig. 4C) activities. Consistent with these findings MBZ elicits anti-oxidant properties by abrogating the DSS-induced increased level of malonyl dialdehyde (MDA), a secondary product of lipid peroxidation, in colitis tissue homogenate (Fig. 4D). These results suggest that the protective effects of MBZ against colitis were at least partially mediated by the induction of anti-oxidant and anti-inflammatory responses.

### Mebendazole reduced fibrosis in colon tissue

**Figure 5:**
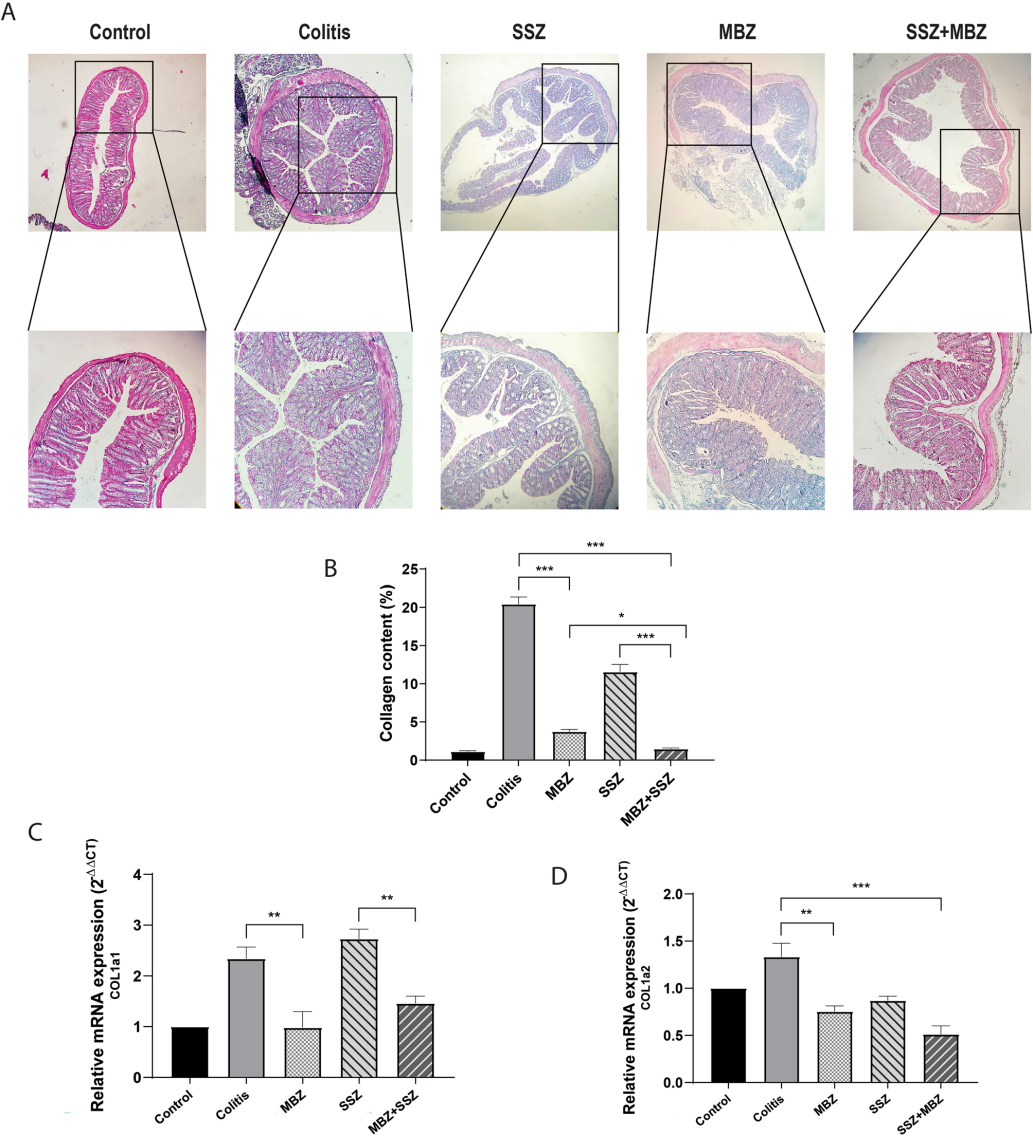
Mebendazole attenuates fibrosis and reduced collagen content. (**A**) Collagen fiber was detected using Masson's trichrome staining. Mebendazole alone or in combination with sulfasalazine significantly reduced collagen deposition. (**B**) Amount of collagen deposition is quantified using Image J software. (**C**–**D**) qRT-PCR results showed the potential effect of MBZ on reducing pro-fibrotic genes including Col1a1 and Col1a2. * *P* < 0.05, ** *P* < 0.01, *** *P* < 0.001. Data were presented as Mean ± SEM, One-way ANOVA followed by post-hoc LSD test. Data are representative of three independent experiments with 6 mice in each group (n = 6).

Colonic wall damage and repair in ulcerative colitis may lead to histological and structural changes including colonic fibrosis^6^. Fibrosis was assessed by quantification of collagen deposition and Masson's trichrome staining. Results showed substantially more collagen deposition in the muscular layer in the DSS-induced colitis group. Administration of MBZ or MBZ + SSZ resulted in less collagen content compared to the untreated colitis model, which was the least in the combination group (Fig. 5A–B). To further investigate the anti-fibrotic effect of MBZ in the colonic tissues, the mRNA expression of fibrotic genes including COL1a1 and COL1a2 was evaluated with quantitative RT-PCR. Our results showed that MBZ significantly reduced tissue expression of these genes compared to colitis mice (Fig. 5C–D). Our findings indicated that MBZ treatment exerted effective anti-fibrogenic effects in colitis-associated intestinal fibrosis.

### Investigating safety of circulating Mebendazole in colitis mice

**Figure 6:**
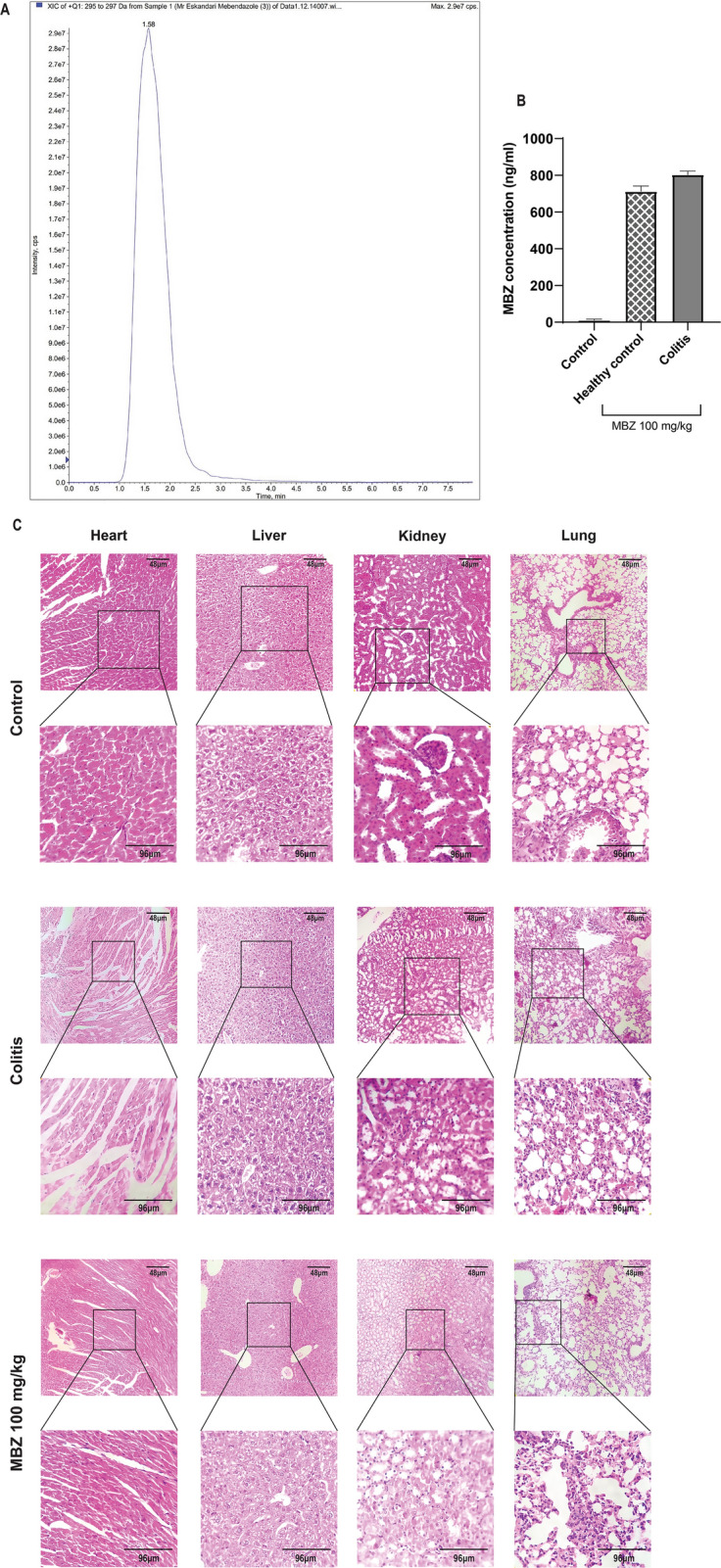
Evaluating the safety of circulating Mebendazole on colitis mice. (**A**) Chromatogram of mebendazole detection by HPLC/MS is shown. Mebendazole was detected with the intensity of 2.9e7 cps. (**B**) Plasma concentrations of mebendazole in no MBZ-treated control, healthy and colitis mice following 100 mg/kg gavage administration of the drug at the day of sacrifice. (**C**) H&E-stained sections of liver, heart, kidney, and lung in control, colitis, and mebendazole groups. Our data showed a normal histological pattern in mebendazole treated mice compared to the control group. Data are representative of three independent experiments with 6 mice in each group (n = 6).

First, the systemic concentrations of MBZ following gavage administration of the drug (100 mg/kg) were measured in healthy and colitis mice using liquid chromatography-mass spectrometry (LC/MS). LC/MS system detected mebendazole in samples with the intensity rate of 2.9e7 cps (Fig. 6A). Our results showed that, on the day of sacrifice, the mean circulating level of MBZ in plasma samples of healthy mice is 712 ng/ml which is increased to 803 ng/ml in colitis mice (Fig. 6B). As is shown, there was a mild but not statistically significant increase in the colitis group.

Next, to determine the safety of this circulating concentration of MBZ, we investigated the effects of MBZ on histopathological changes in the liver, kidney, lung, and heart of treated mice using H&E staining. As is shown in Fig. 3G, gavage administration of MBZ (100 mg/kg) had no pathological effect on these tissues when compared to the no treatment healthy group. Moreover, infiltration of inflammatory cells to the liver and kidney, and disarrangement of myofibers were decreased in MBZ-treated colitis mice when compared to the MBZ–untreated colitis group (Fig. 6C). It is worth noting that, as reported by the National Library of Medicine (Pubchem) the LD50 of MBZ for mice is 620 mg/kg (32)**,** whereas we used 100 mg/kg in this study.

##  Discussion

To the best of our knowledge, this is the first study investigating the therapeutic potential of MBZ on the murine model of UC. Our results showed that the severity of features of colitis was significantly reduced in MBZ-treated colitis mice. We also showed that the protective effects of sulfasalazine, a standard treatment in colitis, could be improved in combination with mebendazole by reducing DAI, histological score, and fibrosis. Protective effects of mebendazole may be attributed to a decrease in inflammatory cell infiltration, histological changes, regulation of oxidant/anti-oxidant ratio, and suppression of collagen deposition.

Ulcerative colitis-induced inflammation is correlated with an acute-phase reaction and a high presence of infiltrated leukocytes to the intestinal tissue, which is one of the most important histological parameters in ulcerative colitis^33^. High levels of leukocytes are thought to release reactive oxygen species (ROS) and secrete pro-inflammatory mediators recruiting inflammatory cells, causing inflammation, and mucosal and histological damages^34,35^. In line with this, Williamson et al*.* showed that MBZ down-regulates the inflammatory markers such as tumor necrosis factor (TNF), IL6, and IL-1β in the C57BL6 *adenomatous polyposis coli* (*APC*)^Min/+^ mice model^21^. Moreover, findings reported that MBZ has potent anti-inflammatory effects which could be related to the ability of mebendazole to deactivate various kinases including extracellular signal-regulated kinase (ERK) and mitogen-activated protein kinase (MEK)^18^, reducing the cyclooxygenase (COX)-2^21^ and TNF-α expression. It also decreased matrix metalloproteinases (MMPs) activities by inducing the tissue inhibitor of matrix metalloproteinases (TIMPs)^19,36^. NF-κB signaling pathway plays a central role in colitis inflammation and is constantly activated in macrophages and other inflammatory cells of colitis patients^37^**.** MBZ potently down-regulates activation of NF-κB signaling by decreasing the translocation of NF-κB complex to the nucleus and p-65 phosphorylation, thus inhibiting inflammation^20^**.** Benzimidazoles including MBZ, are also reported to induce M2-phenotype polarization of macrophages with anti-inflammatory properties, resulting in the production of anti-inflammatory modulators including IL-10 and CD206, and acceleration of the healing process^38^**.** Consistently, our data showed that MBZ either alone or in combination with SSZ can reduce mRNA levels of IL-1β, protein concentrations of TNF-α, the histological changes, and inflammation score in DSS-treated mice.

Furthermore, reactive oxygen species (ROS) contribute to UC pathogenesis by inducing pro-inflammatory responses by activating the NF-κB signaling pathway in macrophages and epithelial cells. This process stimulates the production of TNF-α, IL-6, and IL-1 which enhances leucocytes recruitment leading to tissue damage by further production of ROS^39–41^. High levels of ROS generation can also increase the colitis-associated colorectal cancer risk by local binding to DNA, inducing structural damage. Balanced oxidant/anti-oxidant status may decrease the severity of the disease and could be considered an effective therapeutic target^42^. Here, we evaluated the levels of oxidant and anti-oxidant markers following MBZ treatment. Our results indicate that MBZ alone or in combination with sulfasalazine attenuated oxidative stress marker MDA, increased anti-oxidant activities of CAT and SOD enzymes, and total thiol content in colon tissues.

Fibrosis is another key factor in the pathogenesis of UC. Accumulation of extracellular matrix (ECM) with large amounts of collagen content is a common complication among chronic diseases including colitis^43^. In UC patients, under the stimulation of fibrotic mediators, mesenchymal cells are activated. The activated mesenchymal cells are converted to active myofibroblasts, producing excessive amounts of ECM, exacerbating the injury to bowel walls and fibrosis^6^. Previous data indicated the potency of mebendazole to reduce fibrotic mediators. In a model of pancreatic cancer, Williamson et al. reported that mebendazole could decrease fibrotic content in trichrome-stained tissues accompanied by a reduction in α-SMA^22^. Another study also revealed that MBZ reduced the secretion of ECM proteins such as collagens^23^. Consistently, Masson's trichrome staining showed the remarkable ability of MBZ at decreasing collagen deposition in colitis tissues*.* The protective effects of MBZ alone or in combination with SSZ were more significant compared to SSZ alone. Moreover, our results demonstrated that MBZ could suppress the mRNA expression of fibrotic-associated genes including Col1a1 and Col1a2.

In patients suffering from UC, there are some concerns about the application of drugs because normally bowel wall layers of UC patients are altered, therefore, systemic bioavailability of drugs may increase leading to toxicity^44^. MBZ is normally prescribed in 100 or 200 mg oral tablets twice a day for 3 consecutive days for helminth treatment^45^, but its long-term daily usage is considered a safe therapeutic approach for hydatid cysts and echinococcosis^46,47^. Numerous studies investigated the safety of long-term mebendazole uptake in patients. Results of previous studies on the safety of long-term mebendazole for hydatid cysts showed no toxicity in doses up to 200 mg/Kg/day for 3–12 months, both in children^48^ and adults^16^. Considering probable side effects, Bartoloni et al. reported elevated transaminases in 5% of patients with echinococcosis treated with 50–60 mg/Kg.day for several months^49^. Similarly, Chen et al. showed that gavage administration of MBZ at doses of 50 and 100 mg/kg reduced tumor growth in human myeloma xenografts in nude mice dose-dependently but did not show overt toxicity^25^. It is worth noting that, as reported by the National Library of Medicine (Pubchem), the LD50 of MBZ for mice is 620 mg/kg, whereas we used 100 mg/kg in this study (32). Consistent with these findings, our results showed that gavage administration of mebendazole 100 mg/kg had no histopathological effects on the liver, kidney, lung, and heart and showed no signs of toxicity including weight loss, or mortality in MBZ-treated colitis mice. It is noteworthy that because of the disrupted epithelial barrier in UC patients, there may be some concerns about increased circulating levels of mebendazole after long-term mebendazole administration in patients with chronic or recurrent UC which leads to undesirable side effects and toxicity. Therefore, further research in chronic DSS colitis models should be performed in order to examine extra-intestinal pathologies, circulating MBZ levels, quantitative histopathological changes, and potential side effects to completely demonstrate the safety of mebendazole therapy for patients with UC.

In recent years, due to safety and efficacy, benzimidazoles attract scientists’ attention to be repurposed as alternative therapeutic methods in various diseases. Mebendazole seems to possess protective properties to be repurposed as a new candidate in the treatment of ulcerative colitis, however, it is worth mentioning that in this study we demonstrated a therapeutic effect for MBZ in a single mouse model of ulcerative colitis and further testing in other UC models (e.g., T cell transfer) is needed. In line with this, Wildenberg et al. investigated the therapeutic potency of albendazole, another member of the benzimidazole family, in combination with Infliximab, anti-TNF, in T cell transfer model of colitis. Their findings showed that albendazole potentiated the therapeutic effects of Infliximab by over-expressing the immunoregulatory cytokine IL-10 and by enhancing induction of regulatory macrophages by modulating tubulin skeleton and AMPK signaling in vitro. Consistent with cellular studies, they showed that combination treatment with albendazole and Infliximab was superior to monotherapy in decreasing clinical symptoms and accelerating the healing process in the murine model of colitis^38^. On the other hand, considering the similarities between molecular mechanisms underlying ulcerative colitis and Crohn’s disease, mebendazole may be a good candidate for adjunct therapy in Crohn’s disease as well, but its safety and efficacy must be first investigated in pre-clinical and clinical models of Crohn’s disease.

In conclusion, our study for the first time has shown that the anti-helminth drug mebendazole may be repurposed as a novel therapy for UC treatment. Moreover, combination with SSZ showed better efficacy compared to either treatment alone only in colon length (Fig. 2E), inflammation score (Fig. 3C), MDA concentration (Fig. 4D), and Collagen content (5B). Further investigations in other models of ulcerative colitis and clinical trials are needed to elucidate the exact molecular mechanisms underlying anti-inflammatory and anti-fibrotic properties of the FDA-approved MBZ to be used for patients suffering from UC and other types of IBD in the future.
